# Clinical employment and nursing students’ attitudes towards a humanoid social robot in geriatric care: implications for the public health workforce

**DOI:** 10.3389/fpubh.2026.1925780

**Published:** 2026-09-08

**Authors:** Klaudia Osypka, Malgorzata Dziechciaz, Aleksandra Suwalska, Katarzyna Wieczorowska-Tobis, Slawomir Tobis

**Affiliations:** 1 State University of Applied Sciences in Jaroslaw, Jaroslaw, Poland; 2 Department of Mental Health, Poznan University of Medical Sciences, Poznan, Poland; 3 Geriatrics Unit, Department of Palliative Medicine, Poznan University of Medical Sciences, Poznan, Poland; 4 Department of Occupational Therapy, Poznan University of Medical Sciences, Poznan, Poland

**Keywords:** geriatric care, healthy ageing, humanoid social robot, human-robot interaction, nursing students, technology acceptance, workforce development

## Abstract

**Introduction:**

Population ageing and workforce shortages are placing growing pressure on geriatric care systems, prompting interest in humanoid social robots (HSRs) as a potential support for older-adult care. Their successful introduction, however, depends substantially on acceptance by the nursing workforce that will deliver and oversee their use. Nursing students represent a strategically important population for public health workforce planning, since their openness to robotic technologies will shape how readily such tools are adopted in future practice.

**Methods:**

We conducted a cross-sectional study among 475 nursing students (bachelor’s and master’s level) at a higher education institution in Poland. After viewing a standardised video depicting the TIAGo HSR, participants completed the Users’ Needs, Requirements and Abilities Questionnaire and the Godspeed Questionnaire Series. Students concurrently employed as nurses (*n* = 107) were compared with non-employed students (*n* = 368), and multiple logistic regression was used to adjust for potential confounders.

**Results:**

Overall attitudes were positive, with assistive functions rated more favourably than social functions. Employed students held significantly more favourable views of the robot’s social and companion roles and rated its animacy higher than non-employed peers; professional employment, age, and self-rated technological fluency were independent predictors of attitude.

**Discussion:**

Concurrent nursing employment is independently associated with more favourable, multidimensional attitudes towards HSRs; this cross-sectional association may support the case for embedding human-robot interaction content in nursing curricula and involving frontline nurses in robot implementation, though longitudinal or experimental work is needed to confirm a causal pathway before this is treated as an established public-health-workforce strategy.

## 1 Introduction

The dynamic development of digital technologies, including robotics and artificial intelligence, is remarkably transforming the established healthcare system. In the face of ageing populations, a growing number of chronically ill patients, and medical staff shortages, solutions to support patient care are increasingly being sought (1). One direction of these changes is the development of social robots designed to interact with humans in ways that resemble interpersonal communication. Their primary function is to support relationships, communication, and patient well-being (2). An example of such a robot is the PARO seal, which has been successfully used in non-pharmacological therapy for individuals with cognitive impairment (3). Among these solutions, humanoid social robots (HSRs) occupy a special place. We use “social robot” throughout in its broad, established human-robot-interaction sense - a robot designed to interact with people through natural, human-like communication - which should be distinguished from the narrower “social functions” subscale used later in the UNRAQ (referring specifically to companionship and emotional-support roles, as opposed to the instrumental “assistive functions” subscale). An example of such a robot is Pepper, utilised in care and educational settings (4). Research reports indicate that social robots can reduce feelings of loneliness, support cognitive activation, and facilitate communication with staff (5, 6).

The effectiveness of implementing innovative technologies in healthcare, however, largely depends on the level of their acceptance by medical staff (7). The literature emphasises that the attitude of medical staff towards social robots can be ambivalent. On the one hand, potential benefits are highlighted, such as relieving staff of certain duties, supporting communication with patients, and improving work organisation (8); on the other hand, concerns arise regarding the dehumanisation of care, the limitation of direct human contact, threats to patient privacy, and potential job displacement (9). Even with generally positive attitudes among healthcare professionals towards novel technologies, their limited knowledge may pose a challenge (10).

Nurses, as the professional group in the closest and most direct contact with patients, play a crucial role in implementing new technological solutions in care (11, 12). Their attitudes, beliefs, and level of digital competence can determine both how a social robot is utilised in clinical practice and the quality of care provided to the patient (13). Currently, nursing students, as the future representatives of the profession, constitute a significant research group; their openness to innovation and readiness to work in a technology-supported environment may influence the trajectory of modern nursing development (14, 15).

Despite growing interest in the robotisation of healthcare, the number of studies focusing directly on nursing students’ attitudes towards HSRs remains limited. Understanding their perceptions, expectations, and concerns is essential for technology designers and policymakers responsible for healthcare system organisation and educational curricula. Such knowledge is also directly relevant to the broader goal of sustainable healthcare planning: nursing workforce acceptance of assistive technologies constitutes a prerequisite for their scalable and effective integration into care systems facing mounting demographic pressure.

The aim of this study is to evaluate nursing students’ attitudes towards an HSR and identify the factors influencing these attitudes. A specific aim was to compare attitudes between students with and without concurrent clinical nursing employment, as employment may expose students to the practical realities of older-adult care in ways that classroom-based training alone does not (this aspect has not been considered in our previous studies). The results obtained may serve as a basis for developing recommendations on implementing social robots in nursing practice and modifying educational curricula on digital and technological competencies.

## 2 Materials and methods

### 2.1 Ethical considerations

The study was conducted in accordance with the principles of the Declaration of Helsinki. Participation was voluntary and anonymous, and all respondents provided informed consent prior to enrolment. As this study employed a questionnaire-based methodology, it did not constitute a medical experiment, as formally confirmed by the Bioethics Committee of the Poznan University of Medical Sciences (Approval No. KB-524/25).

### 2.2 Procedure

**Figure 1:**
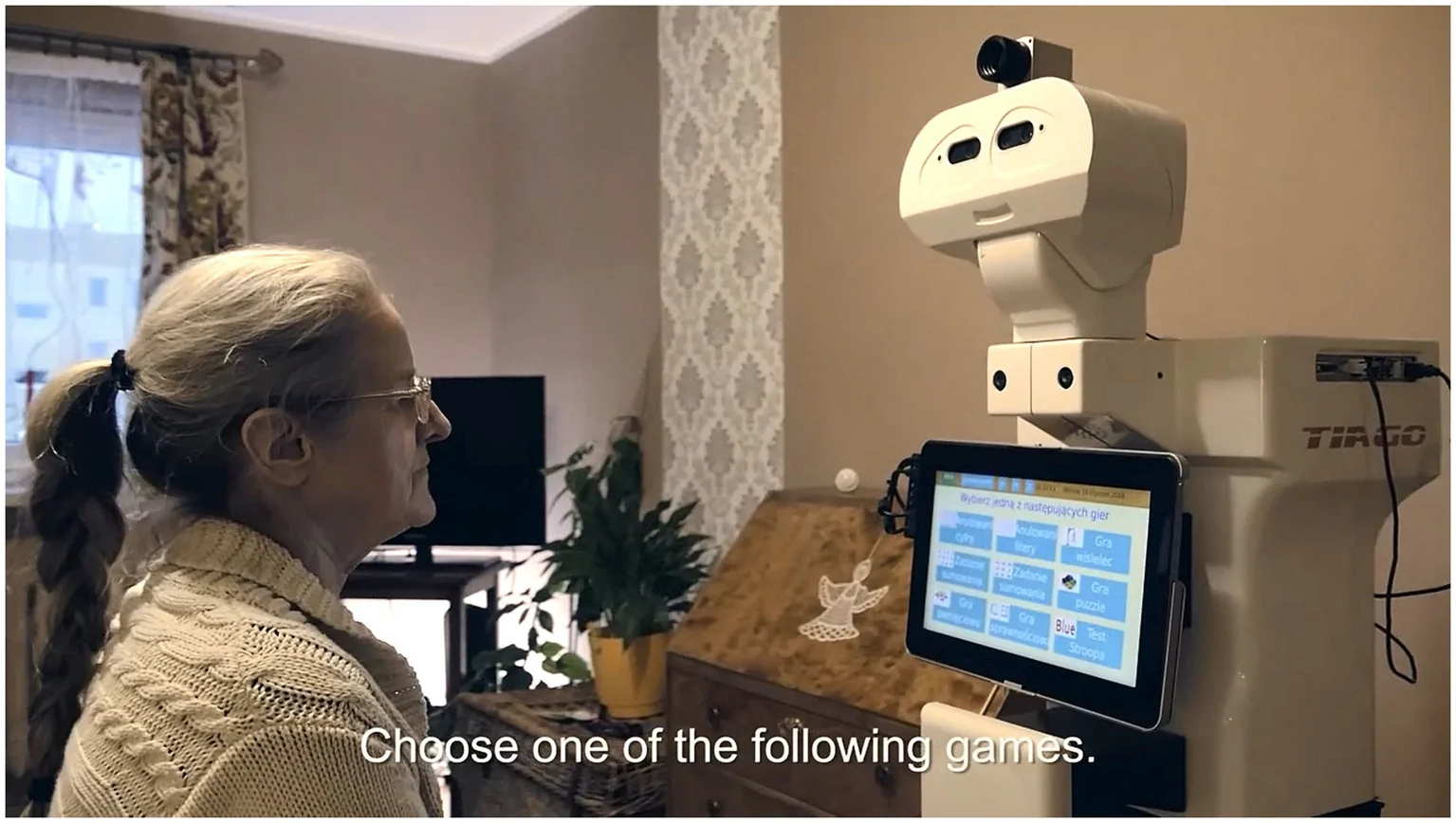
Screenshot from the video used in the instruction phase.

Data collection commenced with an introductory briefing, during which participants were familiarised with the research instruments. Respondents were presented with a short video (Figure 1) depicting a typical day in the life of an older adult assisted by the TIAGo HSR (PAL Robotics, Barcelona, Spain) (16). A comprehensive technical description of the robot’s functionalities has been detailed in previous literature (17). Following the presentation, participants were requested to complete the designated research questionnaires. Unless stated otherwise, all subsequent references to “the robot” in the questionnaire items and throughout this manuscript refer to a broad type of HSRs exemplified by the TIAGo. Participants were asked to consider social robots of the humanoid kind in general.

### 2.3 Participants and setting

The study population comprised nursing students at the State University of Applied Sciences in Jaroslaw, in the Subcarpathian region of Poland (Podkarpacie), recruited during scheduled classes; all who were asked to participate responded positively. The cohort consisted of bachelor’s- and master’s-degree students (the latter held professional nursing diplomas and were already professionally active in healthcare and long-term care settings). Inclusion criteria were defined as:

- Current enrolment in a nursing degree programme.
- Clinical employment (for master’s degree students).
- Provision of informed consent to participate in the study.

### 2.4 Research instruments

The study utilised standardised, validated research tools available in Polish-language versions:

- Users’ Needs, Requirements and Abilities Questionnaire (UNRAQ): this instrument assesses participants’ attitudes towards social robots in care for older adults. Its psychometric robustness has been previously established (18). The questionnaire consists of a socio-demographic section and a main body divided into four thematic areas (domains):
  - Area A – Interaction of the robot and technical issues,
  - Area B – Assistive role,
  - Area C – Social aspects,
  - Area D – Ethical issues.

Responses are recorded on a 5-point Likert scale (ranging from ‘strongly disagree’ to ‘strongly agree’). Results are interpreted by calculating mean scores for individual statements or for the areas as a whole; higher values indicate greater acceptance and perceived utility.

- Godspeed Questionnaire Series (GQS): one of the most widely utilised instruments for evaluating Human-Robot Interaction (HRI), the GQS measures fundamental cognitive and emotional perceptions of robots (19). It comprises five series (subscales):

1. Anthropomorphism,
2. Animacy,
3. Likeability,
4. Perceived intelligence,
5. Perceived safety.

Each series employs a semantic differential format, in which respondents rate the robot on opposing adjective pairs (e.g., ‘mechanical-natural’, ‘unfriendly-friendly’). Each pair is scored on a 5-point scale anchored at its two adjectives, with the first-listed adjective (the more machine-like or negatively valenced pole, e.g., ‘mechanical’, ‘unfriendly’) coded as 1 and the second-listed adjective (e.g., ‘natural’, ‘friendly’) coded as 5, following the standard Godspeed coding convention; a mean score of 2.3 on the mechanical-natural dimension, for instance, indicates a perception weighted towards the mechanical end of that scale, whereas a score of 3.7 would indicate a perception weighted towards natural. Scores are calculated as the mean value of responses within each series. The GQS demonstrates high reliability and construct validity and is considered a benchmark for measuring robot perception in both experimental and applied healthcare settings (20, 21).

The UNRAQ and the GQS were combined because they capture complementary, largely non-overlapping constructs. The UNRAQ assesses context-specific attitudes towards the robot’s roles and functions in older-adult care (e.g., as companion, assistant, or monitoring device), whereas the GQS captures generic, context-independent perceptual and affective dimensions of human-robot interaction (e.g., anthropomorphism, animacy, likeability) that are not tied to a care setting. Used together, the two instruments allow attitudes towards the robot’s care-related utility to be distinguished from more fundamental perceptions of the robot as a social/technical entity.

### 2.5 Statistical analysis

To facilitate quantitative analysis, responses on the Likert scale were assigned numerical values as follows:

- *‘Strongly disagree’* – 1 point
- *‘Partially disagree’* – 2 points
- ‘Neither agree nor disagree’ – 3 points
- *‘Partially agree’* – 4 points
- *‘Strongly agree’* – 5 points

Statistical analyses were conducted using STATISTICA 14.1 (StatSoft, Kraków, Poland) and MedCalc 23.4.9 (MedCalc Software Ltd., Ostend, Belgium). The normality of data distribution was assessed using the Shapiro–Wilk test. Categorical variables were expressed as percentages and frequencies, while continuous variables, due to non-normality, were presented as means ± standard deviation (SD), medians, and interquartile range (IQR).

Given the non-normal distribution of the data, the Mann–Whitney U test was used to compare two independent groups (employed vs. non-employed nursing students). Differences in the distribution of qualitative variables were evaluated using the χ^2^ test, with Yates’s correction applied where appropriate to account for small sample sizes. Individual statements within each area of the UNRAQ were compared using the Friedman analysis of variance (ANOVA), followed by Dunn’s *post hoc* test with standard Bonferroni corrections.

A multiple regression model (logistic regression) was used to assess the simultaneous interdependence among variables, yielding odds ratios (ORs) and 95% confidence intervals (CIs). All variables found to be significant in the univariable analysis were included in the multiple regression analysis, with subsequent backward elimination (22).

For all analyses, a *p*-value of <0.05 was considered statistically significant.

## 3 Results

The mean age of the students included in the analysis (*n* = 475) was 25.7 ± 7.4 years (median 23.0; IQR 20.0–30.0). The sample included only 25 men (5.3%). The characteristics of the study group are presented in Table 1.

**Table 1:** The characteristics of the studied group, including non-working and working students.

Parameter		All students (*n* = 475)	Non-working students (*n* = 368)	Working students (*n* = 107)	Statistical analysis
Age (years)		25.7 ± 7.4 (23; 20–30)	24.9 ± 7.2 (21; 20–29)	29.1 ± 9.6 (24.5; 23–34)	*p* < 0.0001
Sex	Women	447 (94.12%)	350 (95.1%)	97 (90.7%)	ns
	Men	25 (5.3%)	16 (4.3%)	9 (8.4%)	
	Other	3 (0.6%)	2 (0.5%)	1 (0.9%)	
Marital status	Single	314 (66.1%)	253 (68.8%)	61 (57.0%)	*p* < 0.01
	Married/in a relationship	149 (31.4%)	103 (28.0%)	46 (43.0%)	
Living arrangement	Alone	47 (9.9%)	38 (10.3%)	9 (8.4%)	*p* < 0.01
	With spouse	179 (37.7%)	123 (33.4%)	56 (52.3%)	
	With others	249 (52.4%)	207 (56.3%)	42 (39.3%)	
Place of living	Village	358 (75.4%)	282 (76.6%)	76 (71.0%)	ns
	Town	64 (13.5%)	45 (12.2%)	19 (17.8%)	
	City	53 (11.1%)	41 (11.2%)	12 (11.2%)	
Are you familiar with technological systems?		4.3 ± 0.8 (4.0; 4.0–5.0)	4.2 ± 0.8 (4.0; 4.0–5.0)	4.5 ± 0.7 (4.0, 4.0–5.0)	*p* < 0.01
How do you rate your health status?		4.2 ± 0.8 (4.0; 4.0–5.0)	4.2 ± 0.8 (4.0; 4.0–5.0)	4.3 ± 0.7 (4.0; 4.0–5.0)	ns
Do you feel lonely?		4.0 ± 1.3 (5.0; 4.0–5.0)	4.1 ± 1.3 (5.0; 4.0–5.0)	3.9 ± 1.3 (4.0; 3.0–5.0)	ns

Continuous variables as mean ± SD (median; interquartile range, Q1-Q3), and for ordinal variables as the number of participants meeting the criterion (percentage). ns, not significant.

In terms of students’ attitude toward the robot, UNRAQ ratings were, on balance, favourable, though the strength of endorsement varied by domain rather than being uniformly high: mean scores for assistive-role items (area B) were consistently above 4.0, indicating clearly positive endorsement, whereas several items in areas A and C fell in the 3.0–4.0 range, which is more consistent with neutral-to-modestly-positive attitudes than with strong acceptance. Only the statements related to older adults’ ability to operate the robot (A4-A6) scored below the neutral midpoint (i.e., below 3.0) (Table 2).

**Table 2:** UNRAQ results, including the non-working and working students.

Area	Statement	All students (*n* = 475)	Non-working students (*n* = 368)	Working students (*n* = 107)
A. Interaction of the robot and technical issues	A1 The robot should be a companion of the older person	3.3 ± 1.1 (4.0; 3.0–4.0)	3.2 ± 1.2 (3.0; 2.0–4.0)	3.6 ± 1.0** (4.0; 3.0–4.0)
	A2 The robot should be an assistant of the older person	3.5 ± 1.1 (4.0; 3.0–4.0)	3.4 ± 1.2 (4.0;3.0–4.0)	3.8 ± 1.0** (4.0; 4.0–5.0)
	A3 The robot should be a useful device of the older person (something to be used when needed, with no other interaction)	3.9 ± 1.1 (4.0; 3.0–5.0)	3.8 ± 1.2 (4.0; 3.0–5.0)	4.0 ± 0.9 (4.0; 4.0–5.0)
	A4 Older people are prepared to interact with a robot	1.9 ± 1.1 (2.0;1.0–2.0)	1.8 ± 1.0 (2.0; 1.0–2.0)	2.2 ± 1.2** (2.0;1.0–3.0)
	A5 Older people are able to manage with the robot	1.9 ± 1.0 (2.0; 1.0–2.0)	1.9 ± 1.0 (2.0; 1.0–2.0)	2.1 ± 1.1 (2.0; 1.0–3.0)
	A6 Older people want to increase their knowledge about the robots to be able to operate them	2.5 ± 1.2 (3.0; 1.0–3.0)	2.5 ± 1.2 (3.0; 1.0–3.0)	2.7 ± 1.1 (3.0; 2.0–4.0)
	A7 The robot should instruct the older person what to do in case of a problem with its operation	4.3 ± 1.2 (5.0; 4.0–5.0)	4.2 ± 1.2 (5.0; 4.0–5.0)	4.3 ± 1.0 (5.0; 4.0–5.0)
	A8 The robot should be customisable (adjusted to individual user preferences and needs)	4.4 ± 1.1 (5.0; 4.0–5.0)	4.4 ± 1.1 (5.0; 4.0–5.0)	4.4 ± 1.0 (5.0; 4.0–5.0)
	A9 Older people should be able to choose the functions of the robot they want to use and disable other ones	4.0 ± 1.2 (4.0; 3.0–5.0)	3.9 ± 1.3 (4.0; 3.0–5.0)	4.0 ± 1.0 (4.0;4.0–5.0)
	A10 If the robot has been switched off by the owner, it should reactivate automatically (after a specific period) so that it is not forgotten in off mode	4.0 ± 1.2 (4.0; 3.0–5.0)	4.0 ± 1.2 (4.0; 3.0–5.0)	4.1 ± 1.1 (4.0; 4.0–5.0)
B. Assistive role	B1 The robot should increase the safety of the older person’s home: e.g. locking doors, detecting leaking gas etc.	4.3 ± 1.1 (5.0; 4.0–5.0)	4.2 ± 1.1 (5.0; 4.0–5.0)	4.4 ± 1.0 (5.0;4.0–5.0)
	B2 The robot should help older people to preserve their memory function, e.g., by playing memory games with them	4.4 ± 0.9 (5.0; 4.0–5.0)	4.4 ± 1.0 (5.0; 4.0–5.0)	4.4 ± 0.9 (5.0; 4.0–5.0)
	B3 The robot should encourage and guide older people to perform physical exercises	4.4 ± 1.0 (5.0; 4.0–5.0)	4.3 ± 1.0 (5.0; 4.0–5.0)	4.4 ± 0.9 (5.0; 4.0–5.0)
	B4 The robot should provide advice about a healthy diet	4.4 ± 1.0 (5.0; 4.0–5.0)	4.3 ± 1.0 (5.0; 4.0–5.0)	4.4 ± 0.9 (5.0; 4.0–5.0)
	B5 The robot should monitor the environment (temperature, humidity) and suggest air conditioning adjustment or windows opening	4.4 ± 1.0 (5.0; 4.0–5.0)	4.3 ± 1.0 (5.0; 4.0–5.0)	4.4 ± 0.9 (5.0; 4.0–5.0)
	B6 The robot should measure physiological parameters (blood pressure, heart rate, body temperature) of the older person	4.4 ± 1.0 (5.0; 4.0–5.0)	4.4 ± 1.0 (5.0; 4.0–5.0)	4.4 ± 1.0 (5.0; 4.0–5.0)
	B7 The robot should monitor the amount of food and fluid intake of the owner	4.2 ± 1.0 (4.0; 4.0–5.0)	4.2 ± 1.0 (4.0; 4.0–5.0)	4.3 ± 1.0 (5.0; 4.0–5.0)
	B8 The robot should remind older people about appointments	4.4 ± 1.0 (5.0; 4.0–5.0)	4.3 ± 1.0 (5.0; 4.0–5.0)	4.4 ± 0.9 (5.0; 4.0–5.0)
	B9 The robot should remind older people about medication	4.6 ± 0.9 (5.0; 5.0–5.0)	4.6 ± 0.9 (5.0; 5.0–5.0)	4.6 ± 0.8 (5.0; 4.0–5.0)
	B10 The robot should remind about meals times, drinks	4.4 ± 1.0 (5.0; 4.0–5.0)	4.4 ± 1.0 (5.0; 4.0–5.0)	4.5 ± 0.9 (5.0; 4.0–5.0)
	B11 The robot should observe the behaviour of the older person to detect falls or changes due to illness	4.4 ± 1.0 (5.0; 4.0–5.0)	4.3 ± 1.0 (5.0; 4.0–5.0)	4.5 ± 0.9 (5.0; 4.0–5.0)
	B12 The robot should call the centre in case of emergency	4.6 ± 0.9 (5.0; 4.0–5.0)	4.5 ± 0.9 (5.0; 4.0–5.0)	4.6 ± 0.9 (5.0; 5.0–5.0)
	B13 The robot should help the owner to find lost objects (e.g., glasses, keys)	4.3 ± 1.0 (5.0; 4.0–5.0)	4.2 ± 1.1 (5.0; 4.0–5.0)	4.4 ± 0.9 (5.0; 4.0–5.0)
C. Social aspects	C1 The robot could decrease the sense of loneliness and improve the mood of the older person	3.6 ± 1.2 (4.0; 3.0–4.0)	3.5 ± 1.2 (4.0; 3.0–4.0)	3.9 ± 1.1*** (4.0; 4.0–5.0)
	C2 The robot could encourage older people to enhance their contacts with friends	3.9 ± 1.1 (4.0; 3.0–5.0)	3.8 ± 1.2 (4.0; 3.0–5.0)	4.1 ± 0.9** (4.0; 4.0–5.0)
	C3 The robot should initiate contacts with others (calling friends, initiating skype conversations)	3.9 ± 1.1 (4.0; 3.0–5.0)	3.9 ± 1.2 (4.0; 3.0–5.0)	4.2 ± 0.9** (4.0; 4.0–5.0)
	C4 The robot should have entertainment functions (e.g. gaming partner, reading aloud or playing music function)	4.0 ± 1.1 (4.0; 4.0–5.0)	4.0 ± 1.1 (4.0; 3.5–5.0)	4.3 ± 0.9* (4.0; 4.0–5.0)
	C5 The robot should detect the owner’s mood (facial expression)	3.6 ± 1.2 (4.0; 3.0–5.0)	3.5 ± 1.3 (4.0; 3.0–5.0)	4.0 ± 1.0*** (4.0; 4.0–5.0)
	C6 The robot should accompany the owner in everyday activities (watching TV, preparing meals)	3.5 ± 1.3 (4.0; 3.0–4.0)	3.3 ± 1.3 (4.0; 2.0–4.0)	3.9 ± 1.1*** (4.0; 3.0–5.0)
D. Ethical issues	D1 The older person should have control over the robot	4.1 ± 1.1 (4.0; 4.0–5.0)	4.1 ± 1.1 (4.0; 4.0–5.0)	4.0 ± 0.9* (4.0; 4.0–5.0)
	D2 The older person should be able to send the robot to its place/docking station and keep it staying there	4.1 ± 1.1 (4.0; 4.0–5.0)	4.2 ± 1.1 (5.0; 4.0–5.0)	4.0 ± 0.9 (4.0; 3.0–5.0)
	D3 It is acceptable that the robot informs a family member or caregiver about the older person’s behaviour/health problems	4.3 ± 1.0 (5.0; 4.0–5.0)	4.2 ± 1.1 (5.0; 4.0–5.0)	4.4 ± 0.8 (5.0; 4.0–5.0)
	D4 The older person should be able to switch off the robot in specific situations (friends’ visits, privacy reasons etc.)	4.1 ± 1.1 (4.0; 4.0–5.0)	4.1 ± 1.2 (5.0; 4.0–5.0)	4.0 ± 1.1 (4.0; 4.0–5.0)
	D5 It is acceptable that the robot will have much information about the user (social, medical, others)	3.8 ± 1.1 (4.0; 3.0–5.0)	3.7 ± 1.2 (4.0; 3.0–5.0)	3.9 ± 0.9 (4.0; 3.8–4.0)

Continuous variables are presented as mean ± SD (median; interquartile range Q1–Q3), and ordinal variables as the number of participants meeting the criterion (percentage). *p < 0.05; **p < 0.01; ***p < 0.001.

The statement that the robot should be “a useful device of the older person” A3 was rated significantly higher than both being “a companion of the older person” (A1; *p* < 0.001) and “an assistant of the older person” (A2; *p* < 0.001). In addition, being an assistant was rated significantly higher than being a companion (*p* < 0.01).

In the assistive domain, the highest ratings were assigned to statements B9 (“The robot should remind older people about medication”) and B12 (“The robot should call the centre in case of emergency”). These were rated significantly higher than all other items, except for the statement B6 (“The robot should measure physiological parameters [blood pressure, heart rate, body temperature] of the older person”). The lowest score was observed for statement B7, related to monitoring food intake (*p* < 0.05 vs. B5; *p* < 0.01 vs. B2, B10, B11; *p* < 0.001 vs. B6, B9, B12).

In the social domain, functions related to active engagement of older adults, aimed at encouraging interaction beyond the immediate environment (C2-C4), were rated higher than the remaining items (C1 vs. C2: *p* < 0.05; C2 vs. C5: *p* < 0.05; all other comparisons: *p* < 0.001).

The mean score for assistive functions (area B of the questionnaire) was higher than that for social functions (area C) – 4.4 ± 0.9; 4.8 (4.1–5.0) vs. 3.8 ± 1.0; 4.0 (3.3–4.5), (*p* < 0.0001). Moreover, ratings in these two domains were positively correlated (r = 0.6213; *p* < 0.0001).

In the ethical domain, statement D5 (“It is acceptable that the robot will have extensive information about the user (social, medical, and other)”) was rated significantly lower than all other items (*p* < 0.001 for all comparisons).

With regard to the GQS (Table 3), the Anthropomorphism series received the lowest ratings, significantly lower than those of all other series (*p* < 0.01 vs. the Animacy series; *p* < 0.001 vs. the remaining series). Within this series, the items “Machinelike – humanlike” and “Artificial – lifelike” were rated particularly low, scoring significantly lower than “Fake – natural” and “Unconscious – conscious” (all *p* < 0.001).

**Table 3:** Godspeed Questionnaire Series results, including non-working and working students.

Series	Item	All students (*n* = 475)	Non-working students (*n* = 368)	Working students (*n* = 107)
Anthropomorphism	Fake – natural	2.4 ± 1.1 (3.0; 2.0–3.0)	2.4 ± 1.1 (2.0; 1.0–3.0)	2.5 ± 1.0 (3.0; 2.0–3.0)
	Machinelike – humanlike	2.1 ± 1.2 (2.0; 1.0–3.0)	2.1 ± 1.2 (2.0; 1.0–3.0)	2.3 ± 1.1 (2.0; 1.0–3.0)
	Unconscious – conscious	2.5 ± 1.2 (3.0; 1.0–3.0)	2.5 ± 1.2 (2.0; 1.0–3.0)	2.6 ± 1.1 (3.0; 2.0–3.0)
	Artificial – lifelike	2.1 ± 1.1 (2.0; 1.0–3.0)	2.0 ± 1.1 (2.0; 1.0–3.0)	2.3 ± 1.1* (2.0; 1.0–3.0)
	Moving rigidly – moving elegant	2.3 ± 1.2 (2.0; 1.0–3.0)	2.2 ± 1.2 (2.0; 1.0–3.0)	2.5 ± 1.2* (3.0; 1.0–3.0)
	Anthropomorphism – total score	2.3 ± 1.0 (2.2; 1.4–3.0)	2.2 ± 1.0 (2.2; 1.4–3.0)	2.5 ± 1.0* (2.4; 1.6–3.1)
Animacy	Dead – alive	2.4 ± 1.1 (2.0; 1.0–3.0)	2.3 ± 1.2 (2.0; 1.0–3.0)	2.6 ± 1.1* (3.0; 2.0–3.0)
	Stagnant – lively	2.8 ± 1.2 (3.0; 2.0–4.0)	2.8 ± 1.2 (3.0; 2.0–4.0)	2.9 ± 1.1 (3.0; 2.0–4.0)
	Mechanical – organic	2.3 ± 1.2 (2.0; 1.0–3.0)	2.2 ± 1.2 (2.0; 1.0–3.0)	2.6 ± 1.1*** (3.0; 2.0–3.0)
	Artificial – lifelike	2.2 ± 1.1 (2.0; 1.0–3.0)	2.1 ± 1.1 (2.0; 1.0–3.0)	2.4 ± 1.1** (2.5; 2.0–3.0)
	Inert – interactive	3.0 ± 1.3 (3.0; 2.0–4.0)	3.0 ± 1.3 (3.0; 2.0–4.0)	3.1 ± 1.1 (3.0; 2.0–4.0)
	Apathetic – responsive	3.0 ± 1.2 (3.0; 2.0–4.0)	3.0 ± 1.2 (3.0; 2.0–4.0)	3.1 ± 1.2 (3.0; 2.0–4.0)
	Animacy – total score	2.6 ± 1.0 (2.7; 2.0–3.2)	2.6 ± 1.0 (2.5; 2.0–3.2)	2.8 ± 0.9* (3.0; 2.2–3.3)
Likeability	Dislike – like	3.0 ± 1.2 (3.0; 2.0–4.0)	2.9 ± 1.3 (3.0; 2.0–4.0)	3.2 ± 1.0 (3.0; 3.0–4.0)
	Unfriendly – friendly	3.3 ± 1.2 (3.0; 3.0–4.0)	3.3 ± 1.2 (3.0; 3.0–4.0)	3.3 ± 1.1 (3.0; 3.0–4.0)
	Unkind – kind	3.6 ± 1.2 (4.0; 3.0–5.0)	3.7 ± 1.2 (4.0; 3.0–5.0)	3.5 ± 1.1 (4.0; 3.0–5.0)
	Unpleasant - pleasant	3.4 ± 1.2 (3.0; 3.0–4.0)	3.4 ± 1.2 (3.0; 3.0–4.0)	3.4 ± 1.1 (3.5; 3.0–4.0)
	Awful – nice	3.5 ± 1.2 (4.0; 3.0–4.0)	3.5 ± 1.2 (4.0; 3.0–4.0)	3.6 ± 1.1 (4.0; 3.0–4.0)
	Likeability – total score	3.4 ± 1.1 (3.4; 3.0–4.0)	3.4 ± 1.1 (3.4; 2.8–4.0)	3.4 ± 1.0 (3.5; 3.0–4.0)
Perceived intelligence	Incompetent – competent	3.6 ± 1.1 (4.0; 3.0–4.0)	3.5 ± 1.2 (4.0; 3.0–4.0)	3.6 ± 0.9 (4.0; 3.0–4.0)
	Ignorant – knowledgeable	3.5 ± 1.1 (4.0; 3.0–4.0)	3.6 ± 1.2 (4.0; 3.0–4.0)	3.4 ± 1.0* (3.0; 3.0–4.0)
	Irresponsible – responsible	3.5 ± 1.1 (4.0; 3.0–4.0)	3.5 ± 1.1 (4.0; 3.0–4.0)	3.5 ± 1.0 (3.0; 3.0–4.0)
	Unintelligent – intelligent	3.7 ± 1.1 (4.0; 3.0–5.0)	3.7 ± 1.1 (4.0; 3.0–5.0)	3.6 ± 1.1 (4.0; 3.0–4.0)
	Foolish - sensible	3.5 ± 1.1 (4.0; 3.0–4.0)	3.5 ± 1.1 (4.0; 3.0–4.0)	3.5 ± 1.0 (3.0; 3.0–4.0)
	Perceived intelligence – total score	3.6 ± 1.0 (3.6; 3.0–4.2)	3.6 ± 1.0 (3.8; 3.0–4.2)	3.5 ± 0.9 (3.4; 3.0–4.0)
Perceived safety	Anxious - relaxed	3.2 ± 1.1 (3.0; 3.0–4.0)	3.1 ± 1.2 (3.0; 2.0–4.0)	3.3 ± 1.0* (3.0; 3.0–4.0)
	Agitated – calm	3.4 ± 1.1 (3.0; 3.0–4.0)	3.3 ± 1.1 (3.0; 3.0–4.0)	3.4 ± 1.0 (3.0; 3.0–4.0)
	Quiescent - surprised	3.1 ± 1.1 (3.0; 3.0–4.0)	3.1 ± 1.0 (3.0; 2.0–4.0)	3.2 ± 0.9* (3.0; 3.0–4.0)
	Perceived safety – total score	3.2 ± 0.9 (3.0; 3.0–4.0)	3.2 ± 1.0 (3.0; 3.0–3.7)	3.3 ± 0.8 (3.3; 3.0–4.0)

Continuous variables are presented as mean ± SD (median; interquartile range Q1–Q3), and ordinal variables as the number of participants meeting the criterion (percentage). *p < 0.05; **p < 0.01; ***p < 0.001.

The highest scores were observed for the Perceived intelligence series, which was rated significantly higher than all other series (*p* < 0.05 vs. the Likability series; *p* < 0.001 vs. the others). All items within this series received consistently high ratings, with mean values of at least 3.5.

Notably, within the Animacy series, the highest ratings were assigned to “Inert – interactive” and “Apathetic – responsive”, which relate to engagement. These were rated significantly higher than “Dead – alive”, “Mechanical – organic”, and “Artificial – lifelike” (all *p* < 0.001).

### 3.1 Comparison of working and non-working students’ opinions

Among the respondents, 107 stated that they were working as nurses while pursuing their studies. Those who were employed were older (*p* < 0.001), more often in a relationship (*p* < 0.01), and more likely to live with a spouse (*p* < 0.01). They also rated their ease of using technology higher (*p* < 0.01) (Table 1).

In the analysis of the UNRAQ questionnaire (Table 2), working students rated the robot higher in the roles of companion and assistant (A1 and A2; *p* < 0.01), as well as in terms of older adults’ preparedness to operate it (A4; *p* < 0.01). They also assigned higher ratings to all social functions of the robot, as reflected by a higher mean score for area C (*p* < 0.0001). In contrast, ratings of all assistive functions were comparable between groups. Additionally, a significant difference was observed for statement D1 (“The older person should have control over the robot”), which was rated higher by non-working than by working students (*p* < 0.05).

In the GQS analysis (Table 3), working students also rated the Anthropomorphism series higher (*p* < 0.05), driven by higher scores for the items “Artificial – lifelike” (*p* < 0.05) and “Moving rigidly – moving elegantly” (*p* < 0.05). They likewise gave higher ratings to the Animacy series (*p* < 0.05), due to higher evaluations of “Dead – alive” (*p* < 0.05), “Mechanical – organic” (*p* < 0.001), and “Artificial – lifelike” (*p* < 0.01).

The remaining series were rated comparably between groups; however, differences were observed for two items within the *Perceived safety* series (“Anxious – relaxed”: *p* < 0.05; “Quiescent – surprised”: *p* < 0.05). The only parameter rated lower by working nurses was “Ignorant – knowledgeable” within the *Perceived intelligence* series (*p* < 0.05).

In view of the observed differences in socio-demographic characteristics between working and non-working nursing students, a multiple regression analysis using backward elimination was conducted in the subsequent stage of the analysis. Full models were constructed for each parameter of the UNRAQ and the GQS, including all potential independent variables. Final models retained only variables that significantly contributed to explaining the dependent variable’s variance. We thus report the results of calculations for the two questionnaires used.

In the final models, the variables that consistently differentiated the analysed groups (working vs. non-working students) were age, ease of technology use, selected parameters of the UNRAQ questionnaire (except D1), and items from the Animacy series of the GQS (Tables 4, 5). This indicates that, beyond age and ease of technology use, engagement in professional nursing practice was associated with more favourable evaluations of the robot as both a companion and an assistant, as well as more positive perceptions of older adults’ ability to operate the robot. At the same time, working nurses gave higher ratings to the robot’s social functions on the UNRAQ questionnaire and to its animacy on the Godspeed questionnaire.

**Table 4:** The results of multiple regression analyses (with backward elimination) including independent variables of UNRAQ.

Statement	OR (95% CI); *p* value	Nagelkerke R^2^	VIF
A1: The robot should be a companion of the older person	0.77 (0.60–0.99); *p* < 0.05	0.12	2.60
A2: The robot should be an assistant of the older person	0.76 (0.58–0.98); *p* < 0.05	0.13	2.41
A4: Older people are prepared to interact with a robot	0.62 (0.48–0.81); *p* < 0.001	0.16	1.15
C1: The robot could decrease the sense of loneliness and improve the mood of the older person	0.74 (0.58–0.95); *p* < 0.05	0.13	2.48
C2: The robot could encourage older people to enhance their contacts with friends	0.74 (0.57–0.96); *p* < 0.05	0.13	3.15
C3: The robot should initiate contacts with others (calling friends, initiating Skype conversations)	0.71 (0.54–0.93); *p* < 0.05	0.14	2.91
C4: The robot should have entertainment functions (e.g. gaming partner, reading aloud or playing music function)	0.69 (0.52–0.92); *p* < 0.05	0.14	2.43
C5: The robot should detect the owner’s mood (facial expression)	0.70 (0.55–0.89); *p* < 0.01	0.15	2.50
C6: The robot should accompany the owner in everyday activities (watching TV, preparing meals)	0.71 (0.56–0.89); *p* < 0.01	0.15	2.42
Area C (mean)	0.53 (0.35–0.82); *p* < 0.0001	0.15	—
D1: The older person should have control over the robot	1.10 (0.87–1.40); ns	0.11	1.22

The dependent variable is employment status (working vs. non-working students). Overall model fit: Cox & Snell R^2^ = 0.14; Nagelkerke R^2^ = 0.21; Likelihood-ratio test: χ^2^ = 48.5, DF = 12, *p* < 0.0001; Hosmer & Lemeshow test: χ^2^ = 5.24, DF = 8, *p* = 0.73. ns, not significant; VIF, Variance Inflation Factor for individual UNRAQ statements.

**Table 5:** The results of multiple regression analyses (with backward elimination) including Godspeed Questionnaire Series items as independent variables.

Series	Item	OR (95% CI); *p* value	Nagelkerke R^2^	VIF
Anthropomorphism	Artificial – lifelike	0.88 (0.70–1.11); ns	0.12	3.02
	Moving rigidly – moving elegantly	0.82 (0.66–1.01); ns	0.13	2.93
	Anthropomorphism – total score	0.82 (0.64–1.07); ns	0.12	—
Animacy	Dead – alive	0.73 (0.57–0.93); *p* < 0.05	0.14	2.78
	Mechanical – organic	0.73 (0.57–0.93); *p* < 0.01	0.15	2.75
	Artificial – lifelike	0.75 (0.59–0.95); *p* < 0.05	0.14	3.86
	Animacy – total score	0.71 (0.54–0.94); *p* < 0.05	0.13	—
Perceived intelligence	Ignorant – knowledgeable	1.11 (0.87–1.42); ns	0.11	1.34
Perceived safety	Anxious – relaxed	0.87 (0.68–1.11); ns	0.11	1.52
	Quiescent – surprised	0.86 (0.67–1.12); ns	0.11	1.32

The dependent variable is employment status (working vs. non-working students). Overall model fit: Cox & Snell R^2^ = 0.12; Nagelkerke R^2^ = 0.19; Likelihood-ratio test: χ^2^ = 41.7, DF = 10, *p* < 0.0001; Hosmer & Lemeshow test: χ^2^ = 6.54, DF = 8, *p* = 0.59. ns, not significant; VIF, Variance Inflation Factor for individual GQS series.

The analysis produced medium effect sizes (Nagelkerke R^2^, as a proportion-of-variance-explained effect size, ranged from 0.11 to 0.16). Multicollinearity checks for the individual UNRAQ and GQS items yielded Variance Inflation Factors (VIFs) below 5, indicating low, acceptable correlation and no-to-minimal multicollinearity. Cronbach’s α values for the UNRAQ (Table 6) and the GQS (Table 7) showed mostly excellent or good, dependable reliability. The only item with a value below 0.8 was Perceived Safety from the GQS (α = 0.79) – a lower α is more forgivable here since this is a short subscale. Cronbach’s α values for the UNRAQ and the GQS are slightly higher than those obtained in the validation studies of the URAQ (18) and the GQS (23).

**Table 6:** Cronbach’s α values for the individual areas of the UNRAQ.

Area of the UNRAQ	Cronbach’s α
A. Interaction of the robot and technical issues	0.87
B. Assistive role	0.97
C. Social aspects	0.91
D. Ethical issues	0.84

**Table 7:** Cronbach’s α values for the individual series of the Godspeed Questionnaire Series.

GQS series	Cronbach’s α
Anthropomorphism	0.91
Animacy	0.90
Likeability	0.93
Perceived intelligence	0.94
Perceived safety	0.79

## 4 Discussion

Our study evaluated nursing students’ attitudes towards a humanoid social robot (TIAGo) intended for use in the care of older adults, using two tools: the UNRAQ and the Godspeed Questionnaire Series. While overall attitudes were broadly positive across the cohort, a salient finding was the consistently more favourable disposition demonstrated by students currently employed in professional nursing practice compared with those without such experience. These differences merit careful consideration in the context of technology implementation in healthcare settings.

### 4.1 Overall attitudes towards the robot

Across the full sample, students endorsed the robot primarily as a useful device rather than as a companion or social partner, with assistive functions (area B of the UNRAQ) attracting substantially higher ratings than social functions (area C). This hierarchy of perceived utility is consistent with findings in comparable populations, in which healthcare professionals and students alike demonstrate greater comfort with robots fulfilling instrumental, task-oriented functions than with those involving relational or affective dimensions of care (14, 15). Notably, the functions attracting the highest endorsement – medication reminders and emergency alerts – are precisely those areas where failures carry the greatest clinical risk, suggesting that students may conceptualise social robotics primarily through a patient-safety lens. This framing is clinically coherent and may reflect two, not mutually exclusive, readings: first, that students see robotic support as most justified where errors carry the greatest clinical consequence; second, that students regard psychosocial and relational care as inherently human work less suited to robotic delegation - an interpretation broadly consistent with the still-limited and mixed evidence on social robots’ effectiveness for reducing loneliness and social isolation, which are themselves recognised determinants of morbidity and mortality in older adults (6, 24).

Regarding the GQS, the Anthropomorphism series received the lowest ratings across the cohort, suggesting that students perceived TIAGo as a machine rather than ascribing human-like qualities to it (19). Perceived intelligence, by contrast, received the highest ratings. This pattern – functional competence accepted more readily than human-like appearance or behaviour – has been reported elsewhere in the HRI literature and may reflect a pragmatic orientation among healthcare trainees, who evaluate robots on the basis of their capacity to support clinical objectives rather than their resemblance to human interlocutors (23, 25).

### 4.2 Differences between working and non-working students

The most theoretically significant findings of this study concern the systematic differences between students engaged in active clinical employment and those without nursing work experience. Employed students rated the robot more favourably as both a companion (A1) and an assistant (A2), and assigned higher scores to all items within the social functions domain (area C of the UNRAQ), whilst ratings for assistive functions (area B) did not differ significantly between groups. This dissociation is particularly instructive: the two groups converged in their endorsement of the robot’s technical capabilities but diverged specifically with respect to its relational and social potential. This pattern suggests that concurrent nursing employment – independently of age or self-rated technological fluency – is associated with more favourable attitudes towards the social dimensions of robotic care.

One plausible interpretation is that nurses engaged in direct clinical practice encounter, on a daily basis, the psychological burden of loneliness, social withdrawal, and diminished autonomy experienced by older adults in both community and institutional settings (24, 26). This experiential exposure may sensitise them to the potential value of technologies that facilitate social engagement and companionship, even where such technologies cannot replicate the depth of human interaction. The finding that working students were more willing to attribute social and companion roles to the robot aligns with broader evidence suggesting that direct professional experience with older populations increases acceptance of care technologies (8, 11, 27, 28). It is also consistent with the proposition that clinical exposure fosters a more nuanced appreciation of the multidimensional needs of older patients – needs that extend beyond the biomedical into the psychosocial domain (5, 6).

An additional, non-mutually-exclusive interpretation is worth raising: employed students’ more favourable attitudes towards the robot’s social and companion roles could partly reflect first-hand exposure to workload pressures and staffing shortfalls in everyday nursing practice, rather than (or in addition to) genuine enthusiasm for human-robot social interaction. Students who have already experienced the practical strain of unmet relational needs in older-adult care may come to view robotic companionship as a pragmatic, “better-than-nothing” supplement to limited staff time, whereas non-employed students without this exposure may hold a more idealised view of what human caregiving can realistically provide. We flag this as a hypothesis for future longitudinal or qualitative work, rather than as a conclusion supported by our dataset.

Further supporting this interpretation, employed students also assigned higher ratings to item A4, which assessed older adults’ preparedness to interact with a robot. This finding is noteworthy, given that the full sample rated items A4-A6 below the neutral midpoint, thus reflecting widespread scepticism about older individuals’ ability and willingness to engage with robotic technology. That working nurses deviated from this pattern – even if modestly – may reflect first-hand observations of older adults’ adaptability and capacity to engage with novel technologies in clinical settings, observations unavailable to students without such experience.

On the GQS, employed students rated TIAGo significantly higher on the Animacy subscale, particularly on items relating to perceived lifelikeness and the organic quality of movement. It is possible that students with clinical experience bring a different interpretive schema to the observation of robotic behaviour: having witnessed a broader range of human movement and expression in patients, including those affected by neurological, motor, or cognitive impairments, they may apply a more contextualised standard against which to assess the robot’s lifelikeness. Alternatively, clinical experience may reduce the novelty effect of automated or semi-autonomous devices, enabling more nuanced and differentiated perceptual judgements. Both explanations here are speculative and warrant empirical investigation in future work. Still, these perceptions may influence willingness to introduce such robots into patient-facing clinical contexts, insofar as care providers who perceive the robot as more lifelike may be more confident in its capacity to form meaningful interactions with older users. It is notable, however, that even among employed students, the Anthropomorphism series remained the lowest-rated domain, indicating that employment moderates but does not eliminate fundamental perceptual distinctions between humans and robots.

The multiple logistic regression analyses revealed that, after accounting for age and self-rated technological fluency – both of which were significantly higher among employed students – professional employment status remained independently associated with more positive attitudes towards the robot’s social functions and its animacy. This finding is methodologically important: it confirms that the observed group differences cannot be attributed solely to demographic or dispositional confounders. The association between employment status and attitudes towards the robot’s social functions and animacy is not fully explained by age or technological fluency alone. Direct nursing practice may cause more favourable attitudes; yet, self-selection – for example, students who are already more open to technology or more interested in innovative care choosing to work while studying – remains an equally plausible explanation.

The independent contribution of technological fluency to the regression models is noteworthy in itself. Students who rated themselves as more at ease with technology were more likely to be employed. Still, ease of technology use also predicted more favourable attitudes towards the robot, independently of employment. This aligns with the broader technology acceptance literature, which consistently identifies self-efficacy with technology as a determinant of attitudes towards and adoption of novel technological systems in healthcare (7, 13). Taken together, these findings suggest that interventions aimed at increasing technology acceptance among nursing students may benefit from targeting two distinct pathways: building general digital competence and providing structured exposure to real-world clinical environments where technology-supported care is observed and practised.

The regression findings provide a more nuanced and methodologically rigorous account of the between-group differences than the univariable comparisons alone could offer. They indicate that professional employment status is independently associated with attitudes towards HSRs in nursing students, beyond what is explained by age or technological familiarity, and that this association is particularly salient for the social, companion, and patient-readiness dimensions of robot acceptance.

### 4.3 Ethical considerations

Across both groups, the item concerning the acceptability of the robot holding extensive personal data about its user (D5) received the lowest ratings within the ethical area of the UNRAQ. This warrants particular attention, as the successful deployment of social robots in geriatric care is likely to depend upon sustained data collection and sharing between the robotic system, healthcare providers, and family members. Student scepticism regarding data privacy may reflect broader societal concerns about surveillance and data governance, or it may indicate a specific professional ethic of patient confidentiality instilled during nursing education (29, 30). Future studies should explore the nature and sources of these concerns in greater depth, particularly in relation to the regulatory and ethical frameworks governing the use of data in health technologies.

### 4.4 Consequences for the public health and ageing workforce

The challenges motivating this research – demographic ageing, nursing workforce shortages, and the growing burden of chronic disease – are core concerns for public health systems worldwide and are central to the global agenda for healthy ageing. HSRs represent one component of a broader technological response that could help health systems extend high-quality geriatric care to a growing older population, particularly where the nursing workforce is stretched thin. The findings of our study suggest that nursing workforce acceptance of such technologies is associated with two potentially modifiable factors – digital competence and clinical experience (including clinical decision-making) – though the cross-sectional design means this is best read as a hypothesis rather than a demonstrated causal lever: whether curriculum design or structured practice-based learning would actually shift attitudes remains to be tested prospectively. With that caveat, workforce readiness to engage with robotic care technologies may be a worthwhile target for educational and organisational policy aimed at supporting healthy ageing; however, our attitudinal data from a single institution do not directly examine workforce capacity, implementation, or public-health outcomes, and we present this as a direction for future, more directly workforce-focused research rather than an established priority.

### 4.5 Limitations

Several limitations should be acknowledged. First, the sample was drawn from a single higher education institution in the Subcarpathian region of Poland; generalisation of findings to nursing students in other national or institutional contexts should therefore be undertaken with caution. Second, the cross-sectional design precludes causal inference regarding the direction of the association between clinical employment and robot attitudes. Whilst it is plausible that professional experience shapes attitudes, it is equally conceivable that students with more positive predispositions towards technology are more likely to seek employment while studying. Longitudinal studies tracking attitudinal change throughout nurse training would help address this ambiguity. Third, although the UNRAQ and the GQS are validated instruments with well-established psychometric properties (18, 19), the study relied on self-reported measures. Observational or experimental paradigms involving direct robot interaction (instead of the video presentation we used, which allowed standardised presentation across a large sample) may yield complementary insights into the determinants of acceptance behaviour. We also note that commercial research-grade platforms such as TIAGo represent a substantial capital investment, and questions of cost, procurement, and maintenance – not addressed by this study – will ultimately shape whether such robots are feasible for routine deployment in resource-constrained care settings. Fourth, the sample was predominantly female (94.1%), reflecting the gender composition of the nursing profession in Poland but limiting generalisability to male nurses or to healthcare professions with different gender profiles. Fifth, participants evaluated a single robot design (TIAGo), so despite being instructed to view the robot as a representative of a broader class of devices, the findings here may not fully generalise to robots with different form factors. Sixth, employment status was self-reported and, in this sample, is closely tied to enrolment in a master’s-level programme; we thus cannot entirely separate the effect of concurrent employment from that of pursuing graduate education more broadly. An additional limitation is that the study did not capture the extent or nature of clinical exposure obtained through student placements. Thus, students classified as non-employed may nevertheless have had some clinical exposure as part of their education.

### 4.6 Implications

The findings carry practical implications for nursing education and for healthcare system planning. The consistently more positive attitudes of clinically employed students suggest that pedagogical strategies incorporating early and sustained clinical exposure – particularly in settings where social robots or other assistive technologies are deployed – may be effective in fostering technology acceptance. The inclusion of modules on human-robot interaction and the ethical governance of care technologies in nursing curricula is likewise indicated (31–33). For healthcare organisations considering the implementation of social robots, these findings underscore the importance of engaging frontline nurses, including those concurrently pursuing academic study, as active stakeholders in the design, piloting, and evaluation processes. Their professional experience and comparatively favourable attitudes towards robotic assistance position them as valuable advocates for integrating technology into patient care. More broadly, the evidence that clinical experience independently predicts openness to social robotic technology speaks to the importance of practice-based learning as a strategy for building sustainable workforce capacity in the context of technology-supported geriatric care.

It is worth emphasising that acceptance, as measured here, is only one precondition for successful implementation and should not be conflated with usefulness or usability. A robot can be well accepted in principle – rated favourably on attitude scales such as the UNRAQ and GQS – and still fail in practice if it does not fit clinical workflows, is burdensome to operate, or does not address a need that staff or older adults actually have. Educational efforts to build acceptance among the future nursing workforce should therefore be paired with human-centred design and workplace evaluation of specific robots, so that investment in fostering openness to these technologies is matched by investment in ensuring the technologies themselves are genuinely useful and usable in routine care.

## Data Availability

The raw data supporting the conclusions of this article will be made available by the authors, without undue reservation.
